# Randomized phase II study of S-1 dosing schedule for resected colorectal cancer

**DOI:** 10.1186/s12885-015-1476-6

**Published:** 2015-06-03

**Authors:** Chu Matsuda, Mamoru Uemura, Ken Nakata, Tatsushi Shingai, Junichi Nishimura, Taishi Hata, Masakazu Ikenaga, Ichiro Takemasa, Tsunekazu Mizushima, Takeshi Kato, Masataka Ikeda, Masayuki Ohue, Kohei Murata, Junichi Hasegawa, Taroh Satoh, Hirofumi Yamamoto, Mitsugu Sekimoto, Riichiro Nezu, Yuichiro Doki, Masaki Mori

**Affiliations:** 1 Department of Surgery, Osaka General Medical Center, Osaka, Japan; 2 Department of Surgery, Gastroenterological Surgery, Graduate School of Medicine, Osaka University, 2-2 Yamada-oka, Suita City, Osaka 565-0871 Japan; 3 Department of Surgery, Sakai City Hospital, Osaka, Japan; 4 Department of Surgery, Osaka Saiseikai Senri Hospital, Osaka, Japan; 5 Department of Surgery, Osaka Rosai Hospital, Osaka, Japan; 6 Department of Surgery, Kansai Rosai Hospital, Hyogo, Japan; 7 Department of Surgery, National Hospital Organization, Osaka National Hospital, Osaka, Japan; 8 Department of Surgery, Osaka Medical Center for Cancer and Cardiovascular Diseases, Osaka, Japan; 9 Department of Surgery, Suita Municipal Hospital, Osaka, Japan; 10 Department of Frontier Science for Cancer and Chemotherapy, Graduate School of Medicine, Osaka University, Osaka, Japan; 11 Department of Surgery, Nishinomiya Municipal Central Hospital, Hyogo, Japan

**Keywords:** Colorectal cancer, Randomized phase II, S-1, Dose schedule

## Abstract

**Background:**

Postoperative adjuvant chemotherapy for patients with stage III Colorectal cancer (CRC) is now internationally accepted as standard care for improving patient outcomes. The Adjuvant Chemotherapy Trial of S-1 for Colorectal Cancer (ACTS-CC) confirmed the non-inferiority of S-1 to tegafur/urcail/leucovorin in terms of overall survival and disease-free survival in patients with stage III CRC after curative surgery. However, the 6-month completion rate of S-1 treatment was 76.5 % in the ACTS-CC. Therefore, treatment completion remains an unresolved problem.

**Methods/Design:**

A randomized phase II trial was designed to evaluate the efficacy and safety of oral daily administration and alternate-day administration of S-1 as adjuvant chemotherapy in curatively resected stage III CRC. Enrolled patients were assigned to either S-1 daily administration (Arm A) or alternate-day S-1 administration (Arm B). Assigned treatment will start within 8 weeks after surgery. In both arms, S-1 dosing (oral) will be based on body surface area (80 mg/day for body surface area < 1.25 m^2^, 100 mg/day for 1.25–1.5 m^2^, or 60 mg/day for > 1.5 m^2^). In Arm A, S-1 will be administered orally for 28 days, followed by a 14-day rest. Administration will be conducted for 24 weeks from the date of therapy start. In Arm B, S-1 will be administered orally on alternate days for 28 weeks from the date of the start of therapy. After treatment, all patients will be observed without additional therapy unless recurrent lesions or other cancer lesions occur. The primary endpoint is treatment completion rate. Secondary endpoints include 3-year disease-free survival, compliance, and adverse events.

**Discussion:**

Previously, S-1 alternate-day intake maintained the efficacy of chemotherapy while reducing adverse effects for patients with R0-resected stage II/III gastric cancer. Improvement of chemotherapy completion rate for patients with colorectal cancer will lead to an improved patient prognosis. Therefore, a randomized phase II trial has been designed to examine the efficacy of alternate-day versus current standard daily S-1 administration as adjuvant chemotherapy for R0-resected stage III colorectal cancer.

**Trial registration:**

This study was registered on 18 February 2014 with University Hospital Medical Information Network Clinical Trials Registry: UMIN000013185

**Electronic supplementary material:**

The online version of this article (doi:10.1186/s12885-015-1476-6) contains supplementary material, which is available to authorized users.

## Background

Colorectal cancer (CRC) is the second most common cancer in Japan, affecting over 100,000 individuals [1]. The Japanese Society for Cancer of the Colon and Rectum (JSCCR) reported recurrence rates of 3.7 % for stage I disease, 13.3 % for stage II disease, and 30.8 % for stage III [2]. Postoperative adjuvant chemotherapy for patients with stage III CRC is now internationally accepted as standard care for improving patient outcomes. The 2010 JSCCR guidelines recommend four regimens as adjuvant therapy for stage III CRC: i.v. 5-fluorouracil/leucovorin, oral tegafur-uracil/leucovorin, capecitabine, and FOLFOX (5-fluorouracil/leucovorin plus oxaliplatin) [2].

S-1 is an oral anticancer agent containing tegafur, gimeracil, and oteracil potassium in a molar ratio of 1:0.4:1 [3]. The Adjuvant Chemotherapy Trial of S-1 for Colorectal Cancer (ACTS-CC) confirmed the non-inferiority of S-1 to tegafur/urcail/leucovorin in terms of overall survival and disease-free survival in patients with stage III CRC after curative surgery [4]. However, the 6-month completion rate of S-1 treatment was 76.5 % in the ACTS-CC [4]. Therefore, treatment completion remains an unresolved problem.

Previously, S-1 alternate-day intake maintained the efficacy of chemotherapy while reducing adverse effects, and was tolerable for patients with R0-resected stage II/III gastric cancer [5]. Therefore, we planned a multicenter, cooperative, prospective, randomized phase II study to compare daily and alternate-day S-1 administration as postoperative adjuvant therapy for CRC.

## Methods/Design

### Registration of the protocol

This study protocol was registered on the website of the University Hospital Medical Information Network, Japan (protocol ID: UMIN000013185) on 18 February 2014. Details are available at: https://upload.umin.ac.jp/cgi-open-bin/ctr/ctr.cgi?function=brows&action=brows&type=summary&recptno=R000015242&language=J

### Digest of the study protocol

#### Objective

A randomized phase II trial was designed to evaluate the efficacy and safety of oral daily administration and alternate-day administration of S-1 as adjuvant chemotherapy in curatively resected stage III CRC. This study protocol was approved by the Institutional Protocol Review Board of Osaka General Medical Center (the affiliation of the Principal Investigator) and other participating institutions (Additional file 1).

#### Study setting

This study is a multi-institutional, prospective, randomized controlled trial that will begin on 1 April, 2014.

#### Study support

This study is supported by a grant from The Supporting Center for Clinical Research and Education (Osaka, Japan), a nonprofit foundation.

#### Endpoints

The primary study endpoint is treatment completion rate. Secondary endpoints include 3-year disease-free survival, overall survival, compliance, and adverse events defined by the Common Terminology Criteria for Adverse Events v.4.0 [6].

### Eligibility criteria

Patients who received curative resection for histopathologically demonstrated stage III (Japanese Classification of Colorectal Cancer 8^th^ edition [7]) colon or rectosigmoid adenocarcinoma were eligible to participate in this study. The main eligibility criteria were: age 20–80 years, starting chemotherapy within 8 weeks after surgery, having an Eastern Cooperative Oncology Group performance status of 0–1, adequate oral intake, preserved major organ functions, and the ability to provide informed consent.

Patients were excluded if they had a previous history of therapy for stage III CRC (except surgery), previous or concomitant CRC (except carcinoma *in situ*), an active malignancy within 5 years, a history of severe anaphylaxis or allergies to any drug, significant active illness that would preclude protocol treatment, undergoing treatment with fluorocytosine, hepatitis B or C virus infection, or severe mental disease. Pregnant or lactating females were also excluded.

Protocol treatment will begin when the patient fulfills the following criteria: total leukocytes <12,000/mm^3^, neutrophils ≥ 1500/mm^3^, platelets ≥100,000/mm^3^, hemoglobin ≥ 9.0 g/dL, aspartate aminotransferase and alanine aminotransferase < 100 IU/L, total bilirubin < 2.0 mg/dL, and creatinine clearance ≥ 60 mL/min.

### Registration

**Fig. 1:**
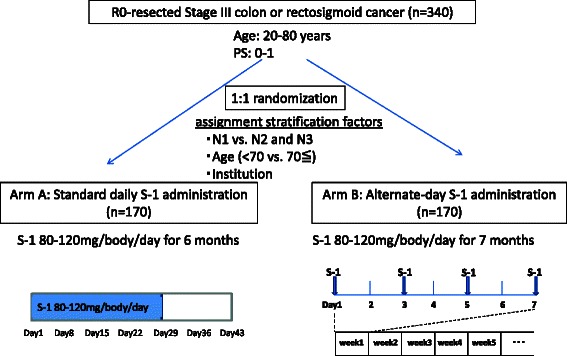
Study design

After confirming eligibility, enrolled patients were randomly assigned to receive either standard daily S-1 therapy (Arm A) or alternate-day S-1 therapy (Arm B) at the MCSGO Data Center. Randomization was performed via a minimization method with stratification by lymph node status (N1 vs. N2 and N3), age (< 70 years vs. ≥ 70 years), and institution (Fig. 1).

### Treatment methods

Enrolled patients were assigned to either S-1 daily administration (Arm A) or alternate-day S-1 administration (Arm B). Assigned treatment will start within 8 weeks after surgery. In both arms, S-1 dosing (oral) will be based on body surface area (80 mg/day for body surface area < 1.25 m^2^, 100 mg/day for 1.25–1.5 m^2^, or 60 mg/day for > 1.5 m^2^).

In Arm A, S-1 will be administered orally for 28 days, followed by a 14-day rest. Administration will be conducted for 24 weeks from the date of therapy start. In Arm B, S-1 will be administered orally on alternate days for 28 weeks from the date of the start of therapy (Fig. 1). After treatment, all patients will be observed without additional therapy unless recurrent lesions or other cancer lesions occur.

In each course, treatment will continue when the patients fulfill the following criteria: total leukocytes ≥ 3000/mm^3^, platelets ≥ 100,000/mm^3^, aspartate aminotransferase and alanine aminotransferase < 100 IU/L, total bilirubin < 2.0 mg/dL, creatinine ≤ 1.5 mg/dL, and diarrhea and stomatitis no greater than grade 1. If the criteria for continuing treatment are not met, then treatment will be postponed or temporarily suspended until the criteria are satisfied.

### Study design and statistical methods

The primary endpoint of this study is treatment completion rate, which is expected to be higher than the treatment completion rate in the ACTS-CC [4] due to alternate-day S-1 administration. Assuming a null hypothesis of 73 % treatment completion and an alternative hypothesis of 83 % treatment completion with one-sided type I error = 0.1 and type II error = 0.2, it was necessary to enroll at least 77 patients in each arm. Assuming 10 % loss to follow-up, we calculated that a total of 170 patients were needed in both treatment arms.

### Decision principle

At the beginning of the study, we established the decision principle to be used after trial results are obtained. If the treatment completion rate for alternate-day S-1 administration (Arm B) is better than that for daily S-1 administration (Arm A), and if the 3-year disease-free survival and adverse-events rates of Arm B is improved or approximately the same as those of Arm A, then alternate-day S-1 administration will be recommended as adjuvant chemotherapy for stage III R0-resected CRC patients.

## Discussion

The feasibility of S-1 treatment as an adjuvant chemotherapy for colorectal cancer was confirmed by ACTS-CC [4], although the protocol treatment completion rate remained 76.5 %. Previously, S-1 alternate-day intake maintained the efficacy of chemotherapy while reducing adverse effects for patients with R0-resected stage II/III gastric cancer [5]. Improvement of chemotherapy completion rate for patients with colorectal cancer will lead to an improved patient prognosis.

Therefore, a randomized phase II trial has been designed to examine the efficacy of alternate-day versus current standard daily S-1 administration as adjuvant chemotherapy for R0-resected stage III colorectal cancer.

## Additional file

Additional file 1: **All Ethics Committees that approved the study.** (16KB)
