# Supplementary material for: Randomized phase II study of S-1 dosing schedule for resected colorectal cancer
Source: BMC Cancer. 2015 Jun 3;15:452. doi: 10.1186/s12885-015-1476-6 (PMC4470361; doi:10.1186/s12885-015-1476-6)
Supplement: Additional file 1: — All Ethics Committees that approved the study. (16KB) [file 12885_2015_1476_MOESM1_ESM.docx]

**All Ethics Committees that approved the study**

Protocol Review Board of Osaka General Medical Center (the affiliation of the Principal Investigator)

The Osaka University Ethics Committee for Research Screening

The Ethics Committee of Sakai City Hospital

The Ethics Committee of National Hospital Organization Kure Medical Center

The Ethics Committee of Osaka Medical Center for Cancer and Cardiovascular Diseases

The Ethics Committee of Suita Municipal Hospital

The Ethics Committee of Nishinomiya Municipal Central Hospital

The Ethics Committee of Kaizuka City Hospital

The Ethics Committee of Toyonaka Municipal Hospital

The Ethics Committee of Rinku General Medical Center

The Ethics Committee of Hannan Chuo Hospital

The Ethics Committee of Yao Municipal Hospital

The Ethics Committee of Itami City Hospital

The Ethics Committee of Kinki Central Hospital

The Ethics Committee of Higashiosaka City General Hospital

The Ethics Committee of Ikeda City Hospital

The Ethics Committee of Kinan Hospital

The Ethics Committee of Nara Hospital Kinki University Faculty of Medicine

The Ethics Committee of Iseikai Hospital

The Ethics Committee of Hyogo Prefectural Nishinomiya Hospital
